# Syringocystadenoma Papilliferum of the Bony External Auditory Canal: A Rare Tumor in a Rare Location

**DOI:** 10.1155/2013/541679

**Published:** 2013-05-23

**Authors:** Anastasija Arechvo, Svajunas Balseris, Laura Neverauskiene, Irina Arechvo

**Affiliations:** ^1^Faculty of Medicine, Vilnius University, Ciurlionio 21/27, 03101 Vilnius, Lithuania; ^2^Department of Ear, Nose and Throat Diseases, Republican Vilnius University Hospital, Siltnamiu 29, 04130 Vilnius, Lithuania; ^3^Patologijos Diagnostika Ltd., P. Baublio 4, 08406 Vilnius, Lithuania

## Abstract

Tumors originating from ceruminous glands are rare lesions of the external auditory canal. The lack of specific clinical and radiological signs makes their diagnosis challenging. We report the case of an exceptionally rare benign tumor, a syringocystadenoma papilliferum (SCAP), in an atypical location in the bony segment of the external auditory canal with uncommon clinical signs. The special traits of the case included the following: the most lateral component of the tumor was macroscopically cystic and a granular myringitis with an obstructing keratin mass plug was observed behind the mass. The clinical, audiological, radiological, and histological characteristics of the neoplasm are consequently presented. Intraoperative diagnosis of the epidermal cyst was proposed. The final diagnosis of SCAP was determined only by histological analysis after the surgical excision. The educational aspects of the case are critically discussed.

## 1. Introduction

Syringocystadenoma papilliferum (SCAP) was first described in the dermatological literature in the beginning of the 20th century as a “naevus syringadenomatosus papilliferus” [1]. Occasionally, this type of tumor may arise in the external auditory canal (EAC). The masses in this location can be a real diagnostic challenge because of a nonspecific clinical presentation and the lack of experience of the clinician or pathologist [2]. SCAP is an extremely rare benign tumor that originates from modified apocrine sweat (ceruminous) glands with extensive papillary growth of epithelial elements down into dermis [3]. In 1894, Haugh described the first ceruminous gland tumor of the cartilaginous segment of the EAC [4]. He proposed the term ceruminoma. Later, Wetli et al. tried to classify these tumors into four groups: adenoma, pleomorphic adenoma, adenoid cystic carcinoma, and adenocarcinoma [5]. According to the World Health Organization, the ceruminous gland neoplasms are currently classified as benign ceruminous adenoma, chondroid syringoma, SCAP and malignant adenocarcinoma, adenoid cystic carcinoma, and mucoepidermoid carcinoma [6]. 

To date, fewer than 150 case reports of ceruminous gland tumors originating in the EAC can be found in the literature [4]. Among them, only 11 cases of SCAP have been described [7]. In this report, we present a case with an atypical clinical presentation. As far as we know, this is the first case of an SCAP occurring simultaneously with chronic granular external otitis and myringitis and a keratin mass plug of the bony part of the EAC. Diagnostic modalities and the problem of differentiating SCAP from other benign and malignant masses of the EAC are also critically discussed. 

## 2. Case Report

The present case illustrates an atypical location for an exceptionally rare disease.

**Figure 1:**
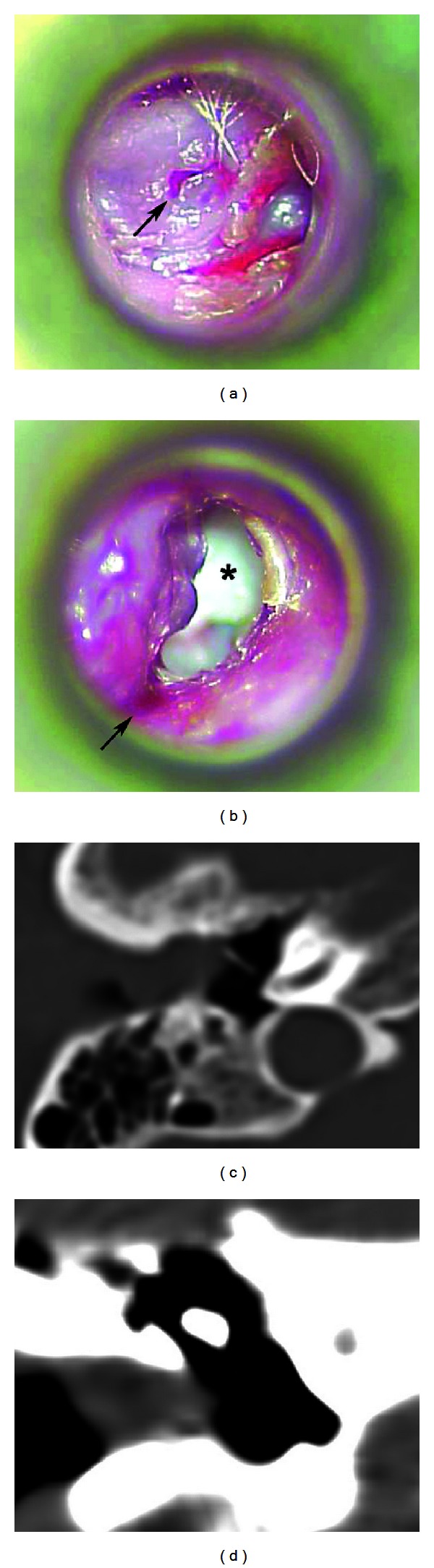
(a) Macroscopic appearance of the EAC polypoid mass with a hole in the center (arrow). (b) When the tumor was elevated, granular tissue was observed at the inferior wall of the EAC (arrow). A large plug of desquamated keratin was observed behind the lesion (asterisk). (c) The soft-tissue mass obstructing the slightly widened bony part of the EAC. (d) A soft-tissue window CT scan revealed the nonhomogenous structure of the tumor.

**Figure 2:**
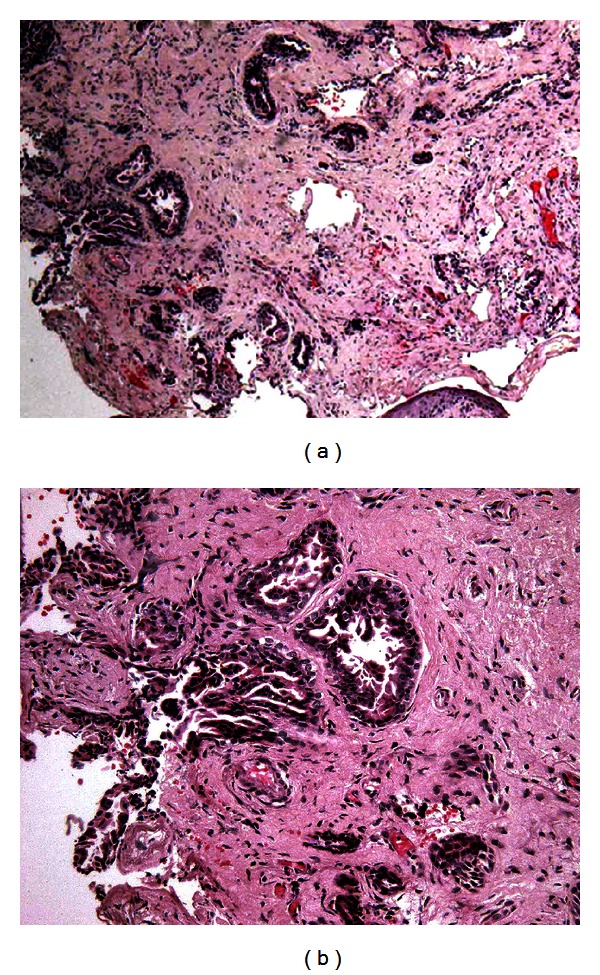
Histological appearance of the tumor, H&E stain. (a) Fragment of dermis with small irregular glands lined by a double-layered epithelium with micropapillary structures and focal squamous metaplasia. (b) Higher magnification of the tumor showing intraglandular micropapillary structures.

**Figure 3:**
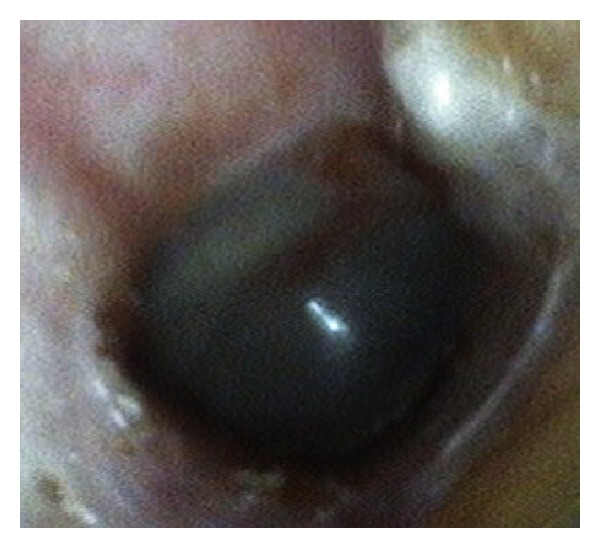
The final postoperative result shows an intact EAC without evidence of tumor recurrence.

A 61-year-old woman presented to the tertiary referral Republican Vilnius University Hospital with a 4-month history of moderate ear pain, fullness, hearing loss, tinnitus, and periodic otorrhea on the right side. She had no previous history of ear infection or discharge. Otoscopically, the bony part of the EAC was fully obstructed by a single pink ulcerated polypoid mass with a hole in the center (Figure 1(a)). Granular tissue at the inferior wall of the EAC was also observed when the tumor was gently elevated with a probe (Figure 1(b)). The mass was painless to touch. Mixed hearing loss up to 53.3 dB with an air-bone gap of 23.3 dB was identified using standard audiometry testing. Tympanometry revealed a B-type curve. A preoperative high-resolution computed tomography (CT) scan of the temporal bones was carried out to evaluate the extension of the mass and differentiate the pathology of the EAC from the more common middle ear diseases. On the CT scan, the middle ear cleft was intact. The bony part of the EAC was slightly widened and filled with the nonhomogeneous soft-tissue mass (Figures 1(c) and 1(d)). Because there was no destruction of the external auditory canal, it was proposed that the tumor was benign in nature, and an excisional biopsy of the lesion under general anesthesia using a transcanal approach was performed. A yellowish liquid and a secretion with a porridge-like consistency leaked from the mass during the procedure. The volume of the tumor decreased, and its origin from the anterior wall of the bony part of the EAC became evident. Keratin masses were observed to accumulate on the anterior tympanomeatal angle. The surface of the intact tympanic membrane was covered by gentle pink granulations. The keratin masses and granulations were removed using a micro dissector and round knife. Then, the tumor was radically removed, and the defects of the ear canal were covered by transposition of the skin flaps. The final histopathological examination revealed a diagnosis of SCAP (Figures 2(a) and 2(b)). The patient was disease-free 9 months after the surgery (Figure 3).

## 3. Discussion

Ceruminous glands are normally located in the outer one-third to one-half of the external auditory canal skin overlying the cartilaginous region. It is generally accepted that the bony canal is devoid of such glands. In the present report, the SCAP originated from the anterior wall of the bony part of the EAC. 

Ceruminous glands tumors have previously been characterized as well-differentiated localized benign neoplasms that are occasionally cystic and demonstrate the papillary proliferation of glands that are histologically similar to normal ceruminous glands [8]. Some authors have proposed a possible relationship between human papillomavirus infection and SCAP [9]. It has been previously reported that SCAP is often associated with a preexisting nevus sebaceous, which is usually located in the head and neck region [10]. No evidence of the nevus or a warty appearance of the tumor, however, was present in our case. Ceruminous gland tumors are uncommon lesions, representing approximately 5% of all tumors of the external auditory canal and auricle [11]. Serious diagnostic difficulties may be encountered because of varied clinical and histological manifestations. SCAP presenting as a soft tissue mass that persist in the EAC should be differentiated from other benign neoplasms, such as cylindroma, papilloma, malignant ceruminous gland tumors, such as squamous cell and basal cell carcinomas, and even tuberculosis [12]. Furthermore, in cases of masses that totally obstruct the canal, only radiological investigations may reveal the exact extent of the mass. The clinical finding of a “polypoid mass” in the EAC in cases of otitis media in up to 13% of cases may present with atypical diagnoses that differ from the traditionally expected diagnosis of middle ear cholesteatoma [13]. 

Occasionally, only histological examination may discover the proper diagnosis. However, when an insufficient amount of material is obtained, the intraglandular papillary structures of an SCAP, for example, may be easily missed. The type of the biopsy and its extent in cases of EAC lesions are still controversial issues. The analysis of similar cases in general does not suggest an exact algorithm for specific diagnoses, treatment opportunities, and clinical outcomes for lesions in the EAC. Preoperative incisional biopsy to establish the diagnosis can be performed to resolve differentiation problems; nevertheless, such biopsies involve a certain level of risk because all ceruminous gland tumors, including SCAPs, are thought to be potentially malignant [14, 15]. In addition, incisional biopsy can cause uncontrollable hemorrhage or facial nerve palsy in cases of glomus tumors or facial schwannomas [7]. Likewise, in the case of obtaining only a small amount of material, a correct pathological diagnosis cannot be determined and may not adequately represent the periphery of the tumor. After excising the lesion, a surgical diagnosis of an epidermal cyst of the EAC was made as the thick fluid (retrospectively, it was determined to be apocrine gland secretion) was aspirated and the volume of the tumor significantly decreased. Furthermore, an epidermoid cyst may exist adjacent to the tumor [6]. If an incisional biopsy was performed, papillae covered by a bilayer of apocrine glandular epithelium could possibly be missed. Because an SCAP contains solid and cystic areas, only the complete removal of the tumor may reveal a definitive diagnosis. SCAP may be a real diagnostic challenge for a pathologist. Significant interobserver variation was recently demonstrated in the diagnosis of SCAP lookalikes [9]. Moreover, squamous epithelium covering the tumor is a nonspecific structure that can also cover cysts, fistulas, and other tumors. It is well known that any obstructing pathology of the external ear can cause accumulation of keratin masses with expansion of the external auditory canal wall, a condition known as keratosis obturans. A case of SCAP occurring simultaneously with a middle ear cholesteatoma and destruction of the long process of the incus has been presented [7]. Based on the present data, we recommend against performing a preoperative incisional biopsy. Other authors have also recommended a wide excisional biopsy for any EAC lesion [2, 4].

High-resolution computed tomography and magnetic resonance imaging are important modalities for diagnosing and differentiating masses in the EAC. Recently, Kamakura et al. presented the MR imaging characteristics of an SCAP of the EAC: intermediate signal intensities on T1- and T2-weighted images and slight enhancement on gadolinium-enhanced T1-weighted images [7]. Judging from the CT images, complete surgical excision can be achieved if there has been no spread into the bony canal and no bone destruction is observed. Following the total removal of the neoplasm, periodic followup is suggested because recurrences may develop in incompletely removed tumors [14].
